# Robot-Assisted Dynamic Interaction of Hemiplegic Upper Limbs with Complex Objects Based on Enhanced Feedforward-Impedance Control

**DOI:** 10.3390/biomimetics10120815

**Published:** 2025-12-04

**Authors:** Jing Bai, Ruoyi Zhu, Yicheng Jiang, Xiaofei Du

**Affiliations:** 1 School of Mechanical Engineering, Nanjing Institute of Technology, Nanjing 211167, China; 18523401932@163.com (R.Z.); 19857215725@163.com (Y.J.); 2 School of Instrument Science and Engineering, Southeast University, Nanjing 210096, China; 3 School of Mechanical and Electrical Engineering, Harbin Engineering University, Harbin 150001, China; duxiaofei@hrbeu.edu.cn

**Keywords:** robot, rehabilitation training, dynamic interaction, feedforward control, sloshing suppression

## Abstract

Current upper-limb rehabilitation robots primarily focus on training tasks involving free movements or static interactions with rigid objects. These paradigms lack simulation of complex object manipulation tasks encountered in daily life, thereby limiting the training of patients’ high-level sensorimotor integration capabilities. To address this gap, this study proposes an innovative robotic rehabilitation training system designed for functional occupational therapy. Specifically, the task of transporting a water cup was abstracted into a cup–ball system integrated with a robotic arm. The ball was modeled as a point mass, and kinematic and dynamic analyses of the system were conducted. A visual tracking method was employed to monitor the ball’s motion and calculate its slosh angle. Owing to the impaired fine motor control in stroke patients, a sloshing suppression control strategy integrating exponential filtering, feedforward force compensation, and impedance control was proposed to prevent the ball from spilling. Experiments validated the effectiveness of the proposed method. The results indicated that with full compensation, the oscillation rate of the ball was significantly reduced, and the smoothness of the hand force was markedly improved. This study presents an effective method for addressing dynamic uncertainty in rehabilitation robot training, thus significantly improving the functional relevance of the training.

## 1. Introduction

The prominent features of stroke include a high incidence rate, high recurrence rate, high disability rate, high mortality rate, and a heavy economic burden [1]. Among survivors of this disease, approximately 75% develop sequelae, of whom 40% have severe disabilities [2]. This severely affects patients’ daily lives and imposes significant suffering and burden on their families.

Among various dysfunctions following a stroke, impairment of upper limb motor function is particularly critical as it directly impacts an individual’s mobility and social participation [3]. The smooth and coordinated execution of fundamental movements such as reaching, grasping, transporting, and precise manipulation forms the cornerstone of performing daily activities like eating and dressing, thereby sustaining personal independence [4]. The improvement of motor function in the hemiplegic upper limb is a vital link in the rehabilitation process.

Conventional rehabilitation training methods, however, are constrained by limitations in intensity, precision, and consistency [5]. In recent years, rehabilitation robotics has emerged as a promising intervention for enhancing motor function by delivering high-intensity, repetitive, and quantifiable training [6,7]. Although existing robotic systems have demonstrated efficacy in facilitating basic motor recovery, their training paradigms predominantly rely on static and decontextualized designs [8]. A significant disparity consequently exists between these paradigms and the dynamic, uncertain task demands characteristic of real-world environments. This discrepancy likely critically constrains the transfer and generalization of rehabilitative gains to activities of daily living.

To address these challenges, this study aims to develop an innovative robotic training system for functional occupational therapy. By introducing dynamic and complex object interaction tasks, this system establishes an ecologically valid training environment, with the objective of more effectively enhancing patients’ functional independence in daily living.

The structure of this paper is as follows: Section 2 reviews the current research on upper limb rehabilitation robots and their control strategies; Section 3 elaborates on the proposed robotic system architecture and core control framework for assisting patients with occupational therapy; Section 4 focuses on simulation and analysis; Section 5 introduces the experimental design and results; Section 6 presents the discussion; finally, Section 7 concludes the paper and outlines future directions.

## 2. State of the Art

This section focuses on analyzing the state of the art in upper-limb rehabilitation robotic training paradigms and their control strategies, while delineating the limitations of existing approaches in simulating the dynamic complexity of the real world.

Current research on upper limb rehabilitation robots mainly focuses on two fundamental task paradigms. The first one is non-contact free-space motion training, where patients operate the robot to complete various trajectory tracking tasks in a virtual environment, such as tracing figures (straight lines, circles, figure eights, etc.) on a screen, or performing free movements along a preset path in three-dimensional space [9,10,11]. The second one is interactive training with rigid objects, including tasks of grasping, lifting, and pushing objects with fixed dynamic characteristics [12,13,14]. These training methods are crucial for restoring the basic range of motion, coordination, and strength of the hemiplegic limbs of patients [15]. However, they create a decontextualized and deterministic training environment, failing to address the core challenges of dynamic uncertainty present in real-life occupational activities.

In terms of control strategies, to achieve safe and effective robot-assisted rehabilitation, researchers have developed various advanced solutions. The impedance/admittance control method has been widely applied to tasks such as manipulating rigid objects and trajectory tracking [16,17]. The integration of impedance control with fuzzy logic or neural networks enables adaptive adjustments based on changes in the patient’s condition, facilitating personalized rehabilitation training [18,19]. Meanwhile, its combination with robust control strategies, such as adaptive sliding mode control, focuses on enhancing the system’s disturbance rejection capability [20]. The incorporation of disturbance observers with adaptive impedance control offers a novel approach for sensorless force control scenarios [21]. Model predictive control methods optimize impedance parameters over a future time horizon, allowing proactive compensation for interactive force disturbances [22,23]. Task-oriented assistance and assessment-driven designs further emphasize the personalization and intelligence of rehabilitation training [24,25]. By decoding motor intentions from bioelectrical signals and integrating them with impedance control, more natural human–robot collaboration can be achieved [26,27]. However, when confronted with the emerging challenge of “dynamic interaction with complex objects”, these strategies reveal inherent limitations. Whether in impedance control or adaptive control, the core focus remains on managing limb movement or regulating the physical interaction between the human and the robot. What is lacking is a mechanism for actively modeling and compensating for the time-varying dynamics of the object being manipulated.

Through a systematic analysis of existing literature, this study identifies core limitations and unresolved key issues in current research when addressing dynamic object interaction tasks: (1) Training paradigms: Existing systems generally lack the capability to effectively simulate dynamic and complex object interaction tasks commonly encountered in daily activities (e.g., carrying a cup containing liquid). This constitutes a significant obstacle in translating current rehabilitation robotics technologies into functional applications. (2) Control strategies: Whether impedance control or adaptive control is employed, the core focus remains on managing the patient’s limb movements or external human–robot interaction forces, while universally neglecting the active modeling and compensation of the internal time-varying dynamics of manipulated objects.

It is noteworthy that significant progress has been made in the field of industrial robotics regarding dynamic tasks such as suppressing liquid sloshing to achieve high-speed and stable transportation [28,29,30]. These studies provide important proof-of-concept for addressing dynamic uncertainties through advanced control methods like model-based feedforward control. While hybrid feedforward-impedance control strategies are established in robotics, their application to occupational therapy tasks, particularly those involving dynamic liquid containers (sloshing), remains largely unexplored. The key novelty of this work is the tailoring and validation of this control paradigm for a therapeutic context.

To address this core challenge, this study proposes an innovative robotic training system for functional occupational therapy. The system utilizes a robotic arm as its platform, with a simplified “cup–ball system” mounted at its end-effector to serve as the physical plant, simulating the dynamic challenges inherent in tasks like “carrying a water cup”. By modeling and compensating for the dynamics of the controlled object, this design aims to assist patients in gradually adapting to and enhancing their perception and precise control capabilities against uncertain dynamics such as liquid sloshing. Ultimately, it seeks to establish a novel paradigm for effectively transferring and generalizing rehabilitation outcomes to real-life scenarios.

## 3. Methods

**Figure 1:**
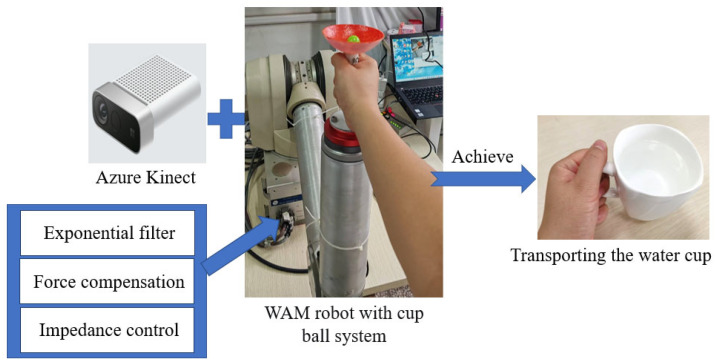
The robotic training system architecture for functional occupational therapy.

The structure of the innovative robotic training system for occupational therapy proposed in this study is illustrated in Figure 1. The system utilizes a WAM (Whole Arm Manipulator, Barrett Technology, Newton, MA, USA) robotic arm as the platform, with a simplified “cup–ball system” (comprising a hemispherical cup and a small ball inside) attached to the end-effector. The motion trajectory of the ball is captured using an Azure Kinect sensor (Microsoft Corporation, Redmond, WA, USA). To simulate the dynamic challenges of “carrying a liquid-filled cup” in occupational therapy, an integrated approach combining exponential filtering, force compensation, and impedance control is employed to suppress ball oscillations. This system assists patients in occupational therapy by progressively enhancing their perception and control capabilities regarding liquid sloshing dynamics.

### 3.1. Mathematical Model of the Cup Ball System

Refer to the transportation coffee experimental platform developed by Sternad et al. [31,32], a hemispherical cup–ball system is designed to enable the robotic arm to assist patients in relearning fine motor skills, specifically the perception-control loop for ball-simulated liquid sloshing, thereby enhancing their functional interaction capacity during daily activities. A small, physical ball is used instead of liquid “coffee”, and the coffee cup is simplified into a hemispherical cup. The bottom of the cup is connected to the mechanical arm through a flange. This setup requires the patient to perform the functional task of moving the cup with robot arm assistance while preventing the ball from falling out, training the ability to dynamically integrate motor commands with sensory feedback.

Assisted by the robotic arm, the subject moves the cup via a handle. A rightward displacement of the cup (along the positive *x*-axis) is defined as positive, and a clockwise swing of the ball along the cup’s inner arc is defined as a positive increase in its slosh angle. Through a resultant force (*F_ext_*) applied to the cup, the robotic arm assists the subject in controlling the cup position (*x_c_*). During the movement, the system directly perceives the interaction force between the ball and the cup. Simultaneously, the arm can employ gravity compensation to counteract the cup’s weight, thereby simulating the real-world activity of carrying a cup of water.

**Figure 2:**
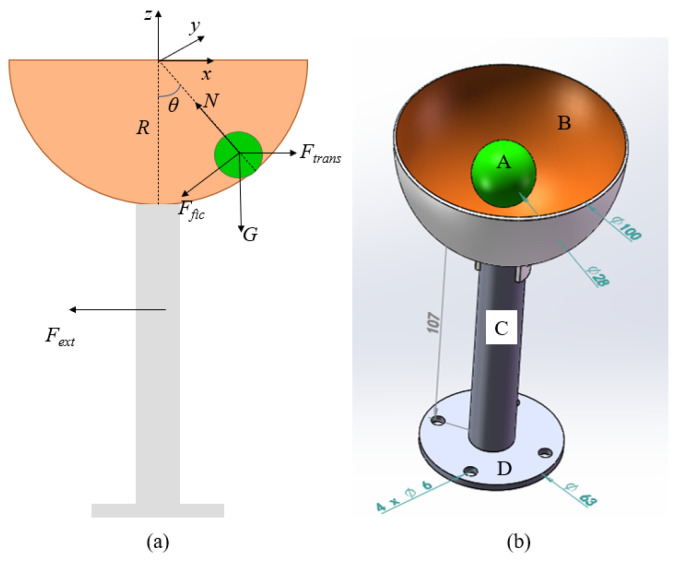
Cup ball system (**a**) Force analysis; (**b**) System 3d model. A is a ball (28 mm diameter), B is a bowl (100 mm diameter), C is a handle (107 mm length), and D is a small hole for connection to a six-axis force sensor with a screw.

The force analysis of the ball-and-cup system is shown in Figure 2a. The ball is primarily subject to the normal force (N) from the cup, gravity (G), friction force (*F_fic_*), and inertial force (*F_trans_*). The three-dimensional model of the system is presented in Figure 2b. Taking the center of the hemisphere cup as the origin, the angle between the normal force acting on the ball and the direction of gravity is *θ*, which characterizes the sloshing motion of the ball. This angle is referred to as the slosh angle of the ball. To simplify the analysis, the motion of the ball is approximated as that of a constrained particle. The mass of the ball is *m*, and a coordinate system is established with the center of the hemisphere as the origin. The position of the ball in the hemisphere cup $r_{b}={\left[x,y,z\right]}^{T}$ is as shown in Equation (1).

(1) $$\left\{\begin{array}{l} x=R\sin\theta \cos\varphi \\ y=R\sin\theta \sin\varphi \\ z=-R\cos\theta \end{array}\right.$$

where *R* is the radius of the hemispherical cup, *θ* represents the polar angle, also referred to as the slosh angle (when *θ* = 0, the ball is at the bottom of the cup, 0 ≤ *θ* ≤ π/2), and *φ* represents the azimuth angle (0 ≤ *φ* ≤ 2π). The kinetic energy of the ball is given by Equation (2). With the center of the hemispherical cup (*z* = 0, corresponding to the ball’s position at *θ* = π/2) set as the zero potential energy reference, the gravitational potential energy of the ball is expressed by Equation (3).

(2) $$T=\frac{1}{2}m({\dot{x}}^{2}+{\dot{y}}^{2}+{\dot{z}}^{2})=\frac{1}{2}mR^{2}({\dot{\theta }}^{2}+\sin^{2}\theta {\dot{\varphi }}^{2})$$

(3) V=mgz=mg(−Rcosθ)=−mgRcosθ

The Lagrangian function is constructed as follows:

(4) $$L=T-V=\frac{1}{2}mR^{2}({\dot{\theta }}^{2}+\sin^{2}\theta {\dot{\varphi }}^{2})+mgR\cos\theta$$

As the cup is accelerated, the ball’s force balance in this co-moving non-inertial frame comprises inertial force effects. The equation for the generalized force balance is expressed as Equation (5).

(5) $$m{\ddot{r}}_{b}=mg+N+F_{fic}-m({\ddot{r}}_{c}+\omega_{c}\times r_{b}+2\omega_{c}\times r_{b}+\omega_{c}\times (\omega_{c}\times r_{b}))$$

where $m{\ddot{r}}_{b}$ represents the inertial force arising from the ball’s relative acceleration within the cup-fixed frame of reference. g is the gravitational acceleration. The non-inertial force term is given as follows: $m{\ddot{r}}_{c}$ is the inertial force caused by the translational acceleration of the cup; $m(\omega_{c}\times r_{b})$ denotes the Euler force arising from the angular acceleration of the cup; $2m\omega_{c}\times r_{b}$ represents the Coriolis force; and $m\omega_{c}\times (\omega_{c}\times r_{b})$ represents Centrifugal force. To simplify the analysis, the rotation of the hemispherical cup is neglected; thus, all terms related to angular velocity disappear.

In the dynamic equation, the constraint force only appears in the normal direction, while the motion in the tangential direction is determined by the applied force. According to D’Alembert’s Principle, the constraint force does no work, so the motion of the ball in the tangential plane of the cup–ball system will not be affected. The tangent plane in spherical coordinates is defined by two unit vectors, $e_{\theta }$ in the direction of increasing *θ*, and $e_{\varphi }$ in the direction of increasing *φ*. These two vectors are orthogonal to each other. We project the motion equation of the ball onto the tangent plane, and the normal constraint force *N* is eliminated, thereby obtaining the tangential motion, which is the equations in the *θ* and *φ* directions. Thus, the original three-dimensional motion problem is transformed into a two-dimensional problem of motion within the tangent plane.

In the spherical coordinate system, variations in the *θ* and *φ* directions precisely describe the motion of the ball on a curved surface. Motion in the *θ* direction corresponds to the ball moving along the meridian, while motion in the *φ* direction corresponds to the ball moving along the parallel. Projecting ${\ddot{r}}_{b}$ onto the $e_{\theta }$ and $e_{\varphi }$ directions:

(6) $$\begin{array}{l} -{\ddot{r}}_{b}e_{\theta }=R(\ddot{\theta }-{\dot{\varphi }}^{2}\sin\theta \cos\theta )\\ -{\ddot{r}}_{b}e_{\varphi }=R(\ddot{\varphi }\sin\theta +2\dot{\varphi }\dot{\theta }\cos\theta ) \end{array}$$

The gravitational and cup acceleration terms are given by Equation (7):

(7) $$\begin{array}{l} (g-{\ddot{r}}_{c})\cdot e_{\theta }=-g(e_{z}\cdot e_{\theta })-{\ddot{r}}_{c}\cdot e_{\theta }\\ (g-{\ddot{r}}_{c})\cdot e_{\varphi }=-{\ddot{r}}_{c}\cdot e_{\varphi } \end{array}$$

We adopt a simple viscous friction model: $f_{f}=-\mu_{t}{\dot{r}}_{b}$, where $\mu_{t}$ is the coefficient of viscous friction. Projecting the frictional force onto the tangential direction:

(8) $$\begin{array}{l} f_{\theta }=-\mu_{t}R\dot{\theta }\\ f_{\varphi }=-\mu_{t}R\sin\theta \dot{\varphi } \end{array}$$

Substituting the above results into the tangential equations yields the dynamic equations in the *θ* direction and *φ* direction, as shown in Equation (9).

(9) $$\begin{array}{l} \ddot{\theta }=\sin\theta \cos\theta {\dot{\varphi }}^{2}+\frac{g}{R}\sin\theta -\frac{a_{c}\cdot e_{\theta }}{R}-\frac{\mu_{t}}{m}\dot{\theta }\\ \ddot{\varphi }=-2\frac{\cos\theta }{\sin\theta }\dot{\theta }\dot{\varphi }-\frac{a_{c}\cdot e_{\varphi }}{R\sin\theta }-\frac{\mu_{t}}{m}\dot{\varphi } \end{array}$$

where $e_{\theta }=\left[\begin{array}{l} \cos\theta \cos\varphi \\ \cos\theta \sin\varphi \\ \sin\theta \end{array}\right],\;e_{\varphi }=\left[\begin{array}{l} -\sin\varphi \\ \cos\varphi \\ 0 \end{array}\right]$, $a_{c}$ represents the acceleration of the cup. When *θ* is very small (that is, when the ball is close to the bottom of the cup), sinθ≈θ, cosθ≈1, the higher-order terms of $\theta^{2}$ and $\theta {\dot{\varphi }}^{2}$ can be neglected. Thus, the dynamic equation can be simplified to Equation (10).

(10) $$\begin{array}{l} \ddot{\theta }\approx \frac{g}{R}\theta -\frac{a_{c}\cdot e_{\theta }}{R}-\frac{\mu_{t}}{m}\dot{\theta }\\ \ddot{\varphi }\approx -\frac{a_{c}\cdot e_{\varphi }}{R\theta }-\frac{\mu_{t}}{m}\dot{\varphi } \end{array}$$

The equation has a singularity when *θ* ≈ 0 in the φ direction. Therefore, the dynamics of the azimuthal angle become unstable when the ball approaches the center of the cup. In practice, however, since the azimuth angle is inconsequential and does not contribute to the ball spilling out when it is near the bottom of the cup, its motion can be neglected. For the radial motion of the ball (in the *θ* direction), an approximate linear equation is obtained as shown in Equation (11), and this mathematical model is equivalent to a general second-order equation.

(11) $$\ddot{\theta }+2\delta \omega_{n1}\dot{\theta }+\omega_{n1}^{2}\theta =-\frac{a_{c}\cdot e_{\theta }}{R}$$

where $\omega_{n1}=\sqrt{g/R},\delta_{1}=\mu_{t}/(2m\omega_{n1})$.

### 3.2. Visual Tracking Algorithm

The trajectories of the ball and the cup are tracked using an Azure Kinect camera, which is installed vertically directly above the ball-and-cup system. The Azure Kinect provides a spatial positioning accuracy within 0.5–2 m and a depth error of less than 2 mm in the optical center region, thereby meeting the precision requirements for tracking the system. Color and depth images of the ball (simulating liquid sloshing) and the cup (at the handle) are captured synchronously. A built-in camera calibration tool from the Kinect SDK is used with a printed checkerboard pattern to establish a mapping between RGB and depth pixels, ensuring one-to-one coordinate correspondence for the same target in both image modalities. RGB images at a resolution of 640 × 580 enable color-based segmentation, while depth images are used to distinguish the target from the background and filter out occlusions.

For accurate tracking of the ball and cup, the interior of the cup is designed to be orange, while the ball is green. Using depth data for image pre-processing, specific Hue, Saturation, Value (HSV) thresholds are defined for both the orange cup and the green ball to generate their respective binary masks, enabling initial extraction of the target regions via RGB color segmentation. Depth data are then utilized to create a region-of-interest (ROI) mask, which excludes background interference such as the patient’s hand and the table edge. The color mask and the depth mask are combined using a logical AND operation to produce a refined mask that contains only the pixels within the target color and depth ranges. Median filtering is applied to the depth image to suppress salt-and-pepper noise inherent in the Kinect depth data. For the resulting fused binary mask, one iteration of erosion with a 5 × 5 kernel is first applied to remove fine noise, followed by two iterations of dilation with the same kernel to restore the original target contours.

Subsequently, target detection and tracking are performed. The contour of the cup is extracted from the fused mask, and an ellipse is fitted using the fitEllipse algorithm to determine the initial center coordinates along with the lengths of the major and minor axes. In the depth image, a 10 × 10-pixel region centered on these coordinates is selected, and the average depth is calculated. By applying a predefined depth threshold, the movement mode of the cup is determined. Based on the elliptical fitting result of the cup, an ROI is delineated in the depth image. Within this ROI, a depth threshold is set to extract pixels with depth values less than or equal to that of the ball, generating a candidate region for ball detection. The HoughCircles algorithm is then applied to detect circular shapes within this candidate region. The detected circles are matched to the actual size of the ball, and those with a radius of approximately 100 mm are selected to identify the ball’s center coordinates.

**Figure 3:**
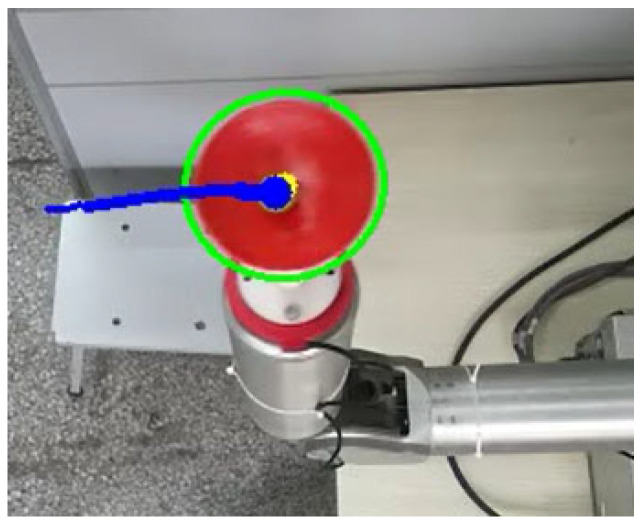
Ball and cup system visual tracking effect. The green ellipse represents the cup contour, the yellow points mark the center of the ball, the blue curve illustrates the trajectory of the ball.

To enhance tracking performance, a Kalman filter is employed for parameter optimization. It utilizes the velocity and acceleration from the previous frame to predict the current position, thereby reducing motion lag caused by low frame rates and improving the stability of depth data. The visual tracking result is shown in Figure 3, where the green ellipse represents the cup contour, the yellow points mark the center of the ball, and the blue curve illustrates the trajectory of the ball.

### 3.3. Sloshing-Suppression-Oriented Enhanced Feedforward-Impedance Control Strategy

**Figure 4:**
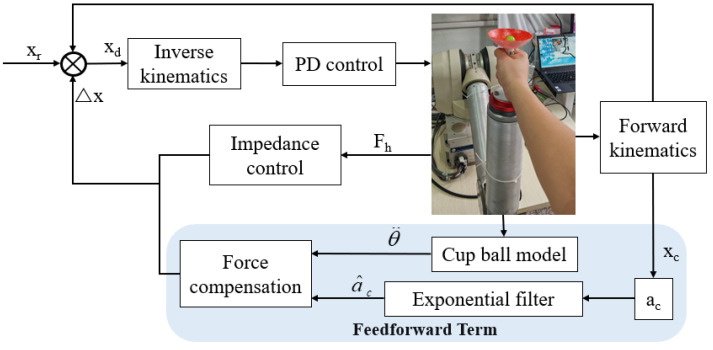
Block diagram of sloshing suppression control strategy, where x_r_ is the desired trajectory, the module enclosed by the dashed line represents the feedforward control, and the impedance can be set as in the equation.

To enable the robotic arm to smoothly assist the subject in transporting the water cup while suppressing sloshing of the ball, an exponential filter is applied to the cup–ball system to smooth the robotic arm’s trajectory, thereby mitigating sloshing dynamics. In addition, a feedforward compensation force is introduced based on impedance control to counteract shaking induced by ball acceleration. Accordingly, this study proposes an enhanced sloshing suppression strategy combining feedforward compensation with impedance control, as illustrated in Figure 4.

The robot employs PD control to provide a stable foundation for low-level feedback, ensuring basic convergence of motion. The exponential filter smooths the reference trajectory of the robotic arm to reduce accelerations that excite sloshing. Based on the dynamic model of the ball, a suitable feedforward compensation force is computed and incorporated into the impedance controller. A six-axis force sensor is employed to measure the interaction force from the patient’s hand. Impedance control ensures compliance in human–robot collaboration. Together, these components enable cooperative human–robot motion that effectively suppresses ball sloshing.

#### 3.3.1. The Exponential Filter

During the robotic arm-assisted movement of the hemispherical cup, the ball inside undergoes oscillations due to inertial forces. The participant must continuously perceive the ball’s motion, predict its dynamic tendency, and adjust their own movement in real time to compensate for the ball’s behavior, thereby preventing it from spilling out of the cup. However, hemiparetic limbs in post-stroke patients are often difficult to control voluntarily. Moreover, movements of the affected limb are frequently accompanied by tremors or sudden jerks—high-frequency noise that transmits through the handle to the cup, exacerbating the sloshing of the ball. Drawing on industrial methods for suppressing liquid sloshing, an appropriate feedforward control action can be applied to attenuate oscillations in such vibratory systems [33]. Dynamic systems exhibiting oscillatory behavior can be modeled by Equation (12), while the structure of the exponential filter is given in Equation (13). Under the conditions of $\sigma =-\delta \omega_{n}$, $T=\frac{2\pi }{\omega_{n}\sqrt{1-\delta^{2}}}$, residual sloshing can be suppressed.

(12) $$G_{0}(s)=\frac{\omega_{n}^{2}}{s^{2}+2\delta \omega_{n}s+\omega_{n}^{2}}$$

(13) $$F_{\exp}(s)=\frac{\sigma }{e^{\sigma T}-1}\frac{1-e^{\sigma T}e^{-Ts}}{s-\sigma }$$

The Laplace transform is applied to both sides of Equation (11), and the transfer function of the ball–hemispherical cup system is obtained as Equation (14).

(14) $$G(s)=-\frac{1}{R}\cdot \frac{1}{s^{2}+2\delta \omega_{n1}s+\omega_{n1}^{2}}$$

The poles of this function are determined by the denominator, as shown in Equation (15). This pair of conjugate complex poles is the inherent vibration mode of sloshing of the ball. Substituting σ and T into $F_{\exp}(s)$, the zero point of the filter is determined by the molecules $1-e^{\sigma T}e^{-Ts}=0$. Simplifying yields $s_{zero}=s_{pole}$. The zero values of the filter and the poles of the system are completely coincident. When the filter is connected in series with the cup–ball system, the zero point of the filter and the poles of the system completely cancel each other out. The sloshing components that would have been amplified/continuously excited by the poles are suppressed/canceled by the zero point of the filter.

(15) $$s_{pole}=-\delta \omega_{n1}\pm j\omega_{n1}\sqrt{1-\delta^{2}}$$

The filtered trajectory is shown in Equation (16).

(16) $$x_{d}(s)=F_{\exp}(s)\cdot x_{ref}(s)$$

where $x_{ref}(s)$ represents the patient’s raw motion trajectory, and $x_{d}(s)$ represents the filtered desired trajectory.

In the frequency domain, the filter’s zeros are used to cancel out the poles of the ball–cup system, thereby blocking the energy transfer at the resonant frequency from the source. In the time domain, the trajectory is smoothed through recursive weighted averaging to achieve a balance between noise suppression and dynamic response speed. This eliminates trajectory discontinuities while not delaying trajectory tracking significantly. The digital control of the robotic arm necessitated the discretization of the continuous transfer function. Discretization via the Zero-Order Hold (ZOH) method resulted in a discrete exponential filter in recursive form, as presented in Equation (17).

(17) $$\left\{\begin{array}{l} Y(n)=\lambda X(n)+(1-\lambda )\cdot Y(n-1)\\ \lambda =1-e^{\sigma T_{s}} \end{array}\right.$$

where *X*(*n*) denotes the raw trajectory value at the *n*-th time step. *Y*(*n*) represents the filtered trajectory value at the *n*-th time step. Based on the foregoing analysis, the exponential filter can perform real-time filtering of the patient’s raw motion trajectory to suppress sloshing of the ball, while providing the patient with perceivable action–stability correlation feedback. This is the core foundation for the subsequent feedforward compensation force and impedance control.

#### 3.3.2. Feedforward Compensation Force and Impedance Control

During robotic-assisted rehabilitation training, the robotic arm is designed to help patients progressively learn to control the cup’s motion to counteract the inertial forces acting on the ball, thereby minimizing sloshing inside the cup. Conventional impedance control in rehabilitation robots typically operates as a feedback strategy, which can only compensate for sloshing after it has been initiated. This reactive approach hinders the establishment of correct movement patterns. Persistent sloshing may degrade the training experience and reduce patient compliance. To address this, the robot can utilize feedforward compensation to allow patients to experience stabilized movement during early training phases. As training progresses, this compensation can be gradually withdrawn to encourage active patient control. By proactively generating a compensation signal that opposes the disturbance, the system can prevent such interference from entering the closed-loop and affecting control performance.

In this study, the feedforward compensation force is derived from the inertial force generated by the ball due to cup motion. Based on the patient’s level of engagement, the robotic arm applies a tailored force to counteract all or part of this inertial force. This approach not only mitigates ball sloshing and prevents spillage but also aids the patient in developing an accurate sense of movement more efficiently.

The raw motion trajectory is processed by exponential filtering to obtain a smooth trajectory, based on which the feedforward compensation force is calculated. The motion of the ball inside the cup is modeled as that of an entrained particle. The force *F_ball_* exerted on the cup is equal in magnitude and opposite in direction to the constraint force exerted by the cup on the ball. The absolute acceleration of the ball consists of the entrainment acceleration due to the cup’s motion and the relative sloshing acceleration of the ball with respect to the cup. Under the small-angle approximation, the tangential sloshing acceleration of the ball relative to the cup is given by $R\ddot{\theta }$. Combining with Equation (11), the feedforward compensation force is derived via Newton’s second law, as presented in Equation (18). The robotic arm acquires joint positions and velocities, computes the cup’s position *x_c_* through forward kinematics, and differentiates it to obtain the raw acceleration *a_c_*. An exponential filter is applied to *a_c_*. A vision sensor detects the ball’s tilt angle *θ* and $\dot{\theta }$ angular velocity in real time, which are substituted into Equation (18) to compute the compensation force *F_comp_*. This force is then combined with the impedance force and sent to the robot controller to actuate the arm.

(18) $$F_{comp}=-F_{ball}=-ma_{b}=-m({\hat{a}}_{c}+R\ddot{\theta })=-m{\hat{a}}_{c}+m{\hat{a}}_{c}e_{\theta }+mR(2\delta \omega_{n1}\dot{\theta }+\omega_{n1}^{2}\theta )$$

In rehabilitation, full feedforward compensation removes all ball sloshing but also eliminates the feedback that conveys “perception and learning”. Without compensation, excessive sloshing makes the task prohibitively difficult, especially for patients with weak limb control who cannot properly maneuver the cup. Therefore, the magnitude of the feedforward compensation force needs to be dynamically adjusted to retain the “perceptible sloshing component for the patient”, allowing the patient to gradually learn to perceive and control the sloshing with the assistance of the robotic arm. We propose an adjustment strategy based on patient engagement level *α* (0 ≤ *α* ≤ 1), where α is calculated from forces measured by a six-axis force/torque sensor, as shown in Equation (19).

(19) $$\left\{\begin{array}{l} F_{comp}(t)=-(1-\alpha )F_{ball}\\ \alpha =\min(\max(\frac{\left\|F_{hand}\cdot n_{slosh}\right\|}{\left\|F_{ball}\right\|},0),1) \end{array}\right.$$

where *n_slosh_* represents the direction vector of the *F_ball_*, ensuring that only the effective component of the human hand’s force along the shake suppression direction is calculated. The denominator corresponds to the theoretical magnitude of the shake force. When *α* = 0, full feedforward compensation is adopted. In this case, the patient only needs to follow the movements of the robotic arm for passive training. When *α* = 1, the patient is active, and there is no compensatory force. When 0 < *α* < 1, *F_comp_* cancels out *F_ball_* according to the patient’s requirements, while the remaining portion must be offset by the patient’s active force exertion. This approach ensures that “perceptible sloshing signals” are preserved to help the patient establish control associations, while simultaneously relying on feedforward compensation to prevent loss of sway control.

By dynamically adapting the feedforward compensation force according to the patient’s degree of engagement, this approach achieves a dual objective: it retains the inherent advantage of feedforward control in disturbance rejection, while simultaneously establishing the crucial “progressive perception-to-control” cycle essential for rehabilitation training. This process enables stroke patients to gradually develop the ability to perceive and regulate liquid-like sloshing vibrations—simulated by the ball’s motion—with robotic arm assistance.

In the context of enhancing compliance control in rehabilitation robotic systems, impedance control is widely recognized as one of the simplest and most effective strategies, which can be implemented via position-based or torque-based frameworks. During rehabilitation training, our goal is to progressively reduce the robotic assistance level as the patient’s motor control improves. The control strategy must therefore balance two key objectives: assisting the patient in completing movements and guiding them to learn autonomous oversuppression of overshoot. Therefore, this study proposes an impedance control method integrated with feedforward compensation. Based on the patient’s engagement level and sloshing indicator, the feedforward compensation force is progressively reduced. The impedance control component enables the robot to exhibit compliant dynamics, while the feedforward compensator proactively counteracts known disturbances or anticipated forces.

Impedance control establishes a dynamic relationship between force and position, allowing the robot’s end-effector to demonstrate compliant mechanical behavior during environmental interaction. The feedforward compensation is designed to counteract the reaction force resulting from ball sloshing within the cup–ball system. This approach helps mitigate dynamic errors in the impedance control, while simultaneously improving response speed and tracking accuracy. The feedforward compensation force is directly injected into the force loop of the impedance controller to preemptively neutralize a portion of the disturbance. This allows the impedance control to primarily respond to the intended interaction force, thereby improving the compliance and naturalness of the human–robot interaction. The force balance equation for the impedance control in Cartesian space is given by Equation (20).

(20) $$F_{cmd}=M_{d}({\ddot{x}}_{d}-{\ddot{x}}_{e})+B_{d}({\dot{x}}_{d}-{\dot{x}}_{e})+K_{d}(x_{d}-x_{e})-F_{h}+F_{comp}$$

where *x_d_* and *x_e_* represent the expected trajectory and the actual end pose of the robotic arm, respectively. *M_d_*, *B_d_*, *K_d_* are, respectively, the target inertia matrix, damping matrix, and stiffness matrix, and *F_cmd_* is the output force of the robotic arm. *F_h_* is the active force measured by the six-axis force sensor of the patient’s hand.

The *F_cmd_* calculated by Equation (20) is the force/torque in Cartesian space. It needs to be converted into torque *τ* in the robot’s joint space to drive the robot’s movement. This transformation is based on the robot’s dynamics model, as shown in Equation (21).

(21) $$M(q)q+C(q,q)q+g(q)=\tau +J_{r}^{T}F_{cmd}$$

where *M*(*q*) is the robot mass matrix, reflecting the inertial properties of the joints; *C* is the Coriolis and centrifugal force vector, representing nonlinear forces during motion; g is the gravity vector, used to compensate for the robot’s own weight; *τ* is the control torque of each robot joint, which is the final signal output to the joint motors; and *J_r_* is the robot Jacobian matrix, which facilitates the transformation between Cartesian space forces/moments and joint space torques.

## 4. Simulation and Analysis

To evaluate the sloshing suppression performance of the feedforward control in the ball–cup system, a double-S trajectory, serving as a simulation of upper-limb reaching motions, was implemented on the rehabilitation robot. A comparative analysis of the slosh angles and force variations with and without feedforward control was conducted. This analysis provides a basis for developing rehabilitation training strategies. The robotic end-effector was commanded to follow this double-S trajectory, moving from the origin along the *x*-axis to the point of full arm extension (0–0.8 m). The mass of the ball was 0.1 kg, the diameter of the cup was 0.1 m, the maximum speed and acceleration were 0.8 m/s and 1.5 m/s^2^, respectively, and the gravitational acceleration was 9.81 m/s^2^.

**Figure 5:**
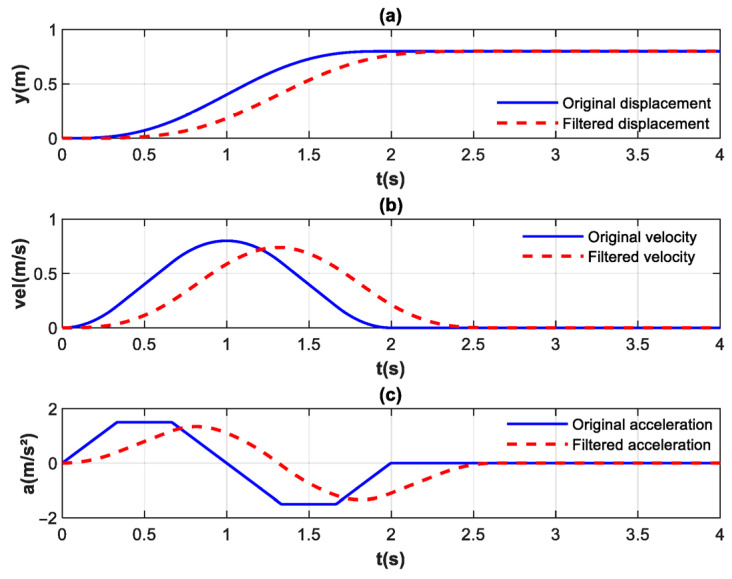
(**a**) Displacement with disturbance and its filtered results. (**b**) Velocity with disturbance and its filtered results. (**c**) Acceleration with disturbance and its filtered results.

Figure 5 illustrates the motion trajectory, velocity, and acceleration of the upper limb. The solid blue lines represent the original motion trajectory, velocity, and acceleration, while the red dashed lines depict the filtered data. A delay is observed in the filtered trajectory, velocity, and acceleration; however, this delay has minimal impact in the context of rehabilitation training. After filtering, the acceleration curve appears exceptionally smooth.

**Figure 6:**
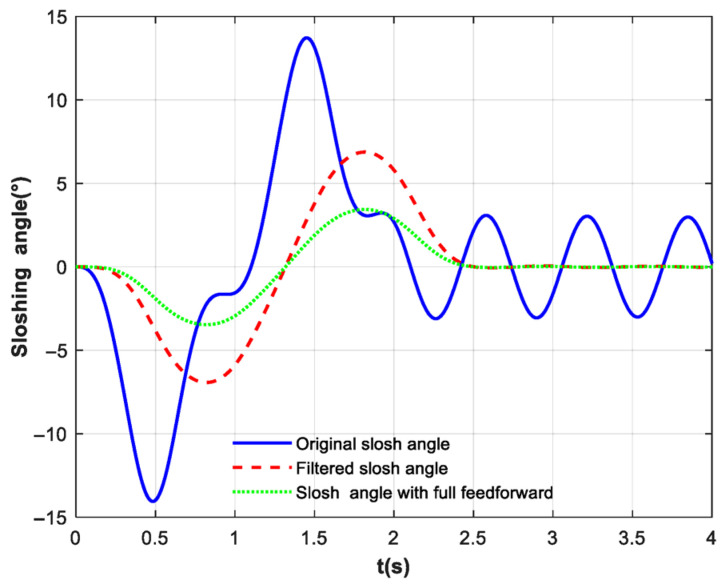
Slosh angle response with ramp disturbance. The blue solid line represents the result without feedforward control. The red dashed line shows the result with the exponential filtering. The green dotted line represents the result obtained with a complete feedforward control strategy.

Figure 6 illustrates the sloshing amplitude of the ball. The blue solid line represents the result without feedforward control, where the maximum slosh angle reaches 14.05°. The red dashed line shows the result with the incorporation of exponential filtering, reducing the maximum slosh angle to 6.93°. The green dotted line corresponds to the result obtained using the complete feedforward control strategy, which further suppresses the maximum slosh angle to 2.77°. A direct comparison of the slosh angles across these three scenarios clearly demonstrates the superior slosh suppression performance of the complete feedforward control method, which combines filtering and compensation force. The achieved sloshing suppression rate for the ball is 80.28%.

**Figure 7:**
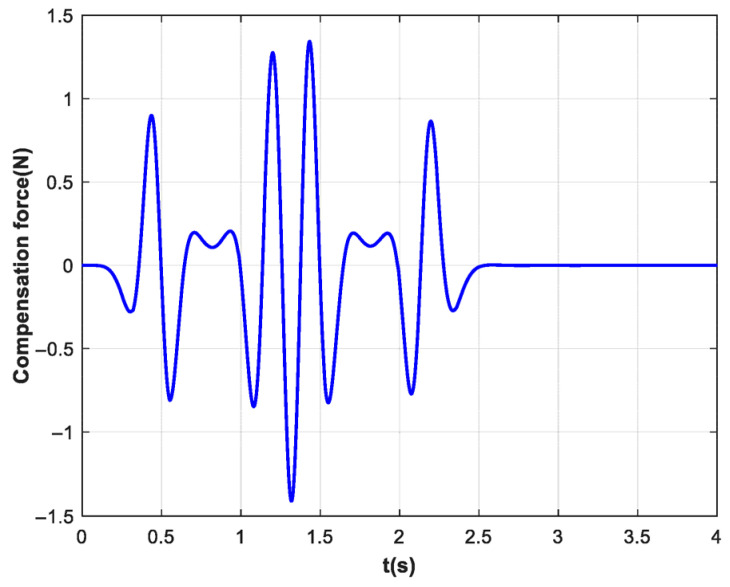
The feedforward compensation force. The compensation force waveform reflects the dynamic coupling of the cup acceleration and ball sloshing.

Figure 7 illustrates the profile of the feedforward compensation force. The simulation validates that the proposed integration of exponential filtering and feedforward force effectively attenuates ball sloshing. Consequently, the robotic system achieves improved perception of human intent and external disturbances, leading to more natural interaction without compromising the safety guaranteed by impedance control.

## 5. Experiment and Result Analysis

### 5.1. Experimental Setup

**Figure 8:**
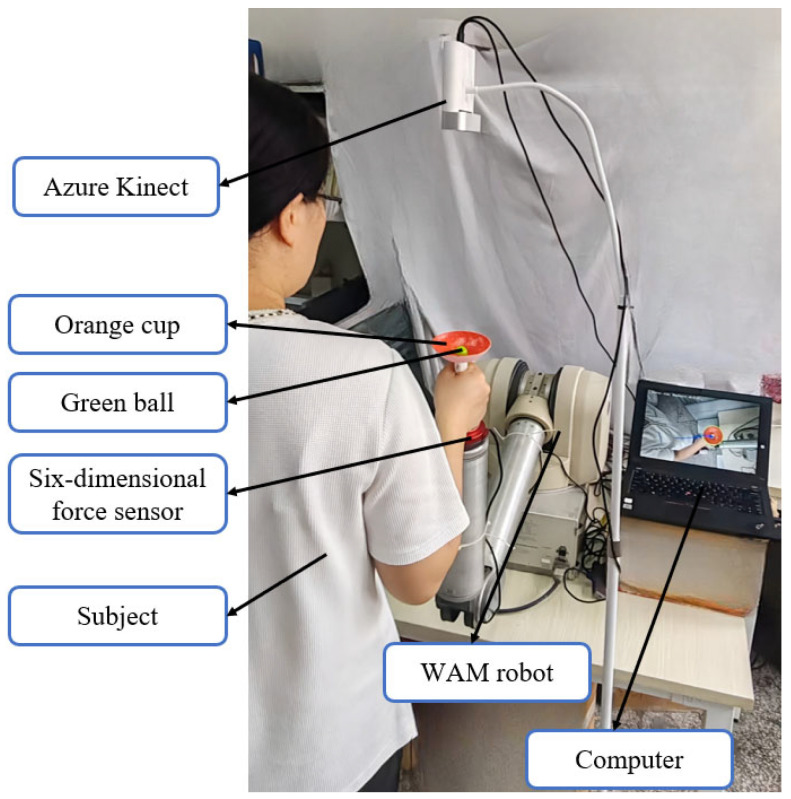
The experimental setup includes a computer, a WAM robot, a six-axis force sensor, a green ball, an orange cup, and an Azure Kinect sensor. A participant is shown performing the experimental task.

The experimental platform is equipped with a four-degree-of-freedom WAM robotic arm produced by the US company Barrett. It employs a direct cable-driven joint mechanism, is back drivable, and communicates with an external PC via CAN bus for data transmission. The experimental setup is shown in Figure 8. A six-axis force sensor is installed between the robotic arm end-effector and the handle to monitor three-dimensional forces (F_x_, F_y_, F_z_) and three-dimensional moments (M_x_, M_y_, M_z_) in real time. It detects both the operating forces applied by the patient and dynamic load variations caused by ball sloshing. The force signals acquired by the six-axis force sensor are filtered using a second-order Butterworth low-pass filter, as expressed in Equation (22).

(22) $$H(s)=\frac{\omega_{c}^{2}}{s^{2}+\sqrt{2}\omega_{c}s+\omega_{c}^{2}}$$

where *ω_c_* is the cut-off frequency. Prior to the experiment, the zero-drift of the force sensor was sampled at 15 Hz while the robotic arm was held static for 10 s. This recorded drift value was then subtracted from the subsequent data to compensate for static error.

The effectiveness of the method proposed in the paper was measured by the sloshing rate of the ball and the rate of change in the force on the subject, as shown in Equation (23).

(23) $$\left\{\begin{array}{l} \eta =\frac{\theta_{\max A}-\theta_{\max B}}{\theta_{\max A}}\times 100\%\\ \gamma =\frac{{\bar{F}}_{\max A}-{\bar{F}}_{\max B}}{{\bar{F}}_{\max A}}\times 100\% \end{array}\right.$$

where η represents the sloshing rate of the ball; $\theta_{\max A}$ represents the maximum value of the original slosh angle; $\theta_{\max B}$ represents the maximum value of the slosh angle after adding the control method; γ represents the rate of change in the force on the subject; ${\bar{F}}_{\max A}$ represents the average maximum value of the raw force; and ${\bar{F}}_{\max B}$ represents the average maximum value of the force after adding the control method.

To facilitate patient gripping, the threaded hole on the six-axis force sensor was connected to the small hole on the flange beneath the cup handle via a screw. The cup–ball system features a bottom-mounted handle connected to the lowest point of the upper cup. This assembly was modeled in SolidWorks (2023) and fabricated from lightweight 3D-printed materials. The cup is colored orange, and the small, rigid green ball has a diameter of 2 cm. The Azure Kinect camera was mounted 0.6 m above the cup’s rim, oriented downward to capture both RGB and depth video streams at a frame rate of 30 fps. A checkerboard pattern is employed for calibration to eliminate lens distortion and intrinsic parameter errors. Synchronization across RGB, depth, and skeletal data is achieved using the hardware timestamp from the Kinect, preventing temporal misalignment between modalities. All data are transmitted via USB 3.0 to a processing unit and stored as pandas DataFrames in a Python (3.10) environment. The *ω_n_* was derived based on the cup radius as 14 rad/s, and the *δ* was experimentally identified as 0.008. The sampling period was set to 1 ms to accommodate high-frequency control for the WAM robotic arm. The six-axis force sensor sampled at 2 kHz, providing high-bandwidth data for the control loop.

During the upper limb rehabilitation period for stroke patients, the upper limb muscle strength is generally weak. A mass of 0.1 kg closely matches the weight of a “small cup of liquid” in daily life. It is neither too heavy to exceed the patient’s initial load-bearing capacity, nor too light to result in insufficient inertial force, which would make it difficult for the patient to perceive the ball’s oscillation. The ball is made of stainless steel with a diameter of 28 mm.

A total of six healthy participants (3 males and 3 females) were recruited for this study. Their baseline physiological characteristics were as follows: age range 22–35 years, mean age 28.5 ± 3.2 years (mean ± SD); height 181.3 ± 3.5 cm for male participants and 165.8 ± 2.8 cm for female participants, with an overall mean height of 173.1 ± 5.2 cm. The inclusion criteria consisted of: no history of neurological disorders or motor dysfunction; no upper limb surgeries, fractures, or muscle injuries within the past 6 months; absence of chronic conditions such as hypertension or diabetes that may affect motor control; and normal vision and hearing with the ability to clearly understand experimental instructions.

Prior to the experiment, the researcher thoroughly explained the study objectives, procedures, potential risks, and data usage to all participants. After ensuring full comprehension, written informed consent was obtained from each participant. They were also informed that they could withdraw from the study at any stage without providing a reason, and that this would not affect their rights or interests. The median results of the rehabilitation training of the six healthy individuals were selected as the outcome of this experiment.

At the beginning of each trial, the ball was placed at the bottom of the cup. The subject faced the display screen and held the WAM robot handle with one hand. At the starting position, the arm is flexed, and at the ending position, the arm is extended. The subject stretched and flexed the arm back and forth between the starting point and the finish line, ensuring that the small ball did not spill out of the cup. This enables upper limb rehabilitation training and enhances the subject’s perception and control ability of liquid sloshing. Experimental scheme: The robotic arm carries a hemispherical cup ball system and performs a reciprocating motion along the *Y*-axis to simulate the action of transporting water cups in rehabilitation training. The motion trajectory adopts the cubic B-spline trajectory commonly used by robots.

### 5.2. Experimental Results

**Figure 9:**
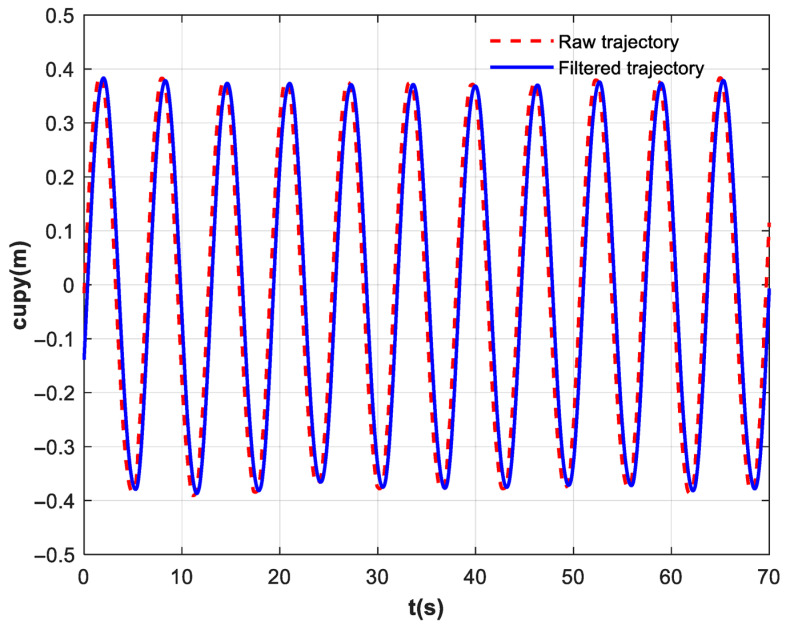
The trajectory of the cup. The red dashed line denotes the raw trajectory, and the blue solid line represents the filtered one.

**Figure 10:**
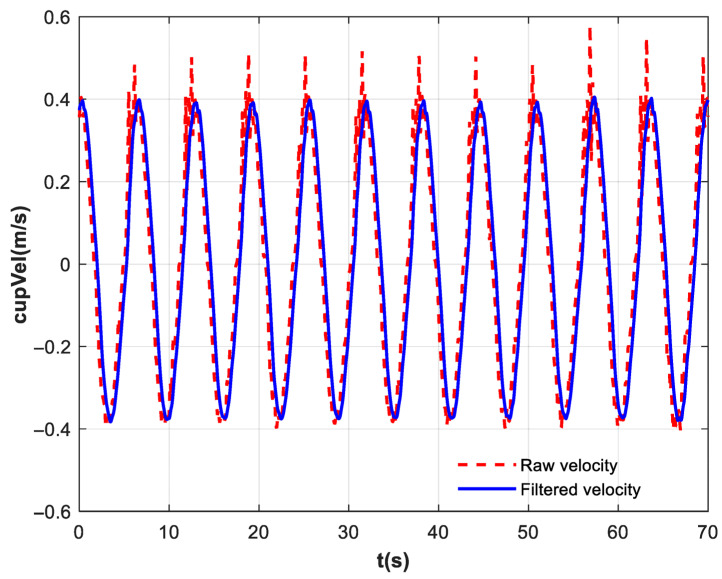
The velocity of the cup. The red dashed line denotes the raw velocity, and the blue solid line represents the filtered one.

**Figure 11:**
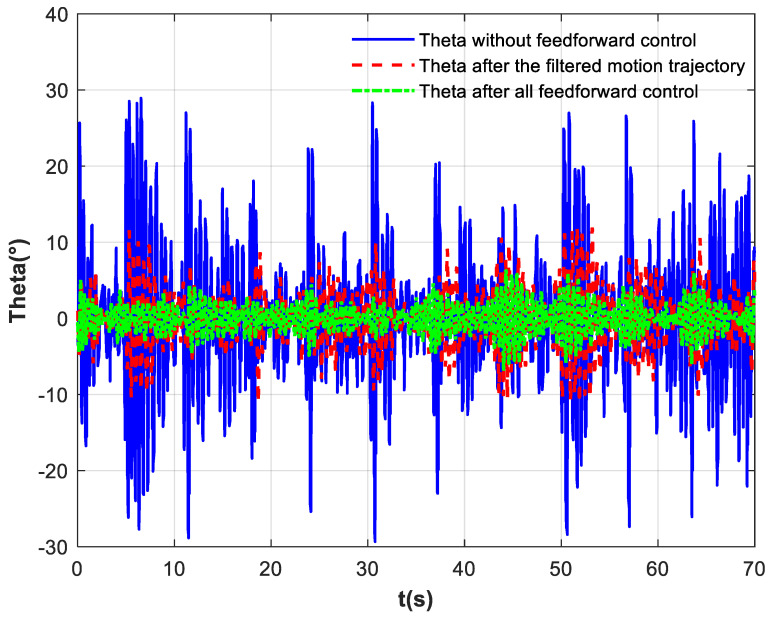
The experimental results of the slosh angle. The blue solid line represents the sloshing angle without feedforward control (A1), the red dashed line represents the sloshing angle with filtered feedforward (A2), and the green centerline represents the sloshing angle with the complete feedforward control (A3).

**Figure 12:**
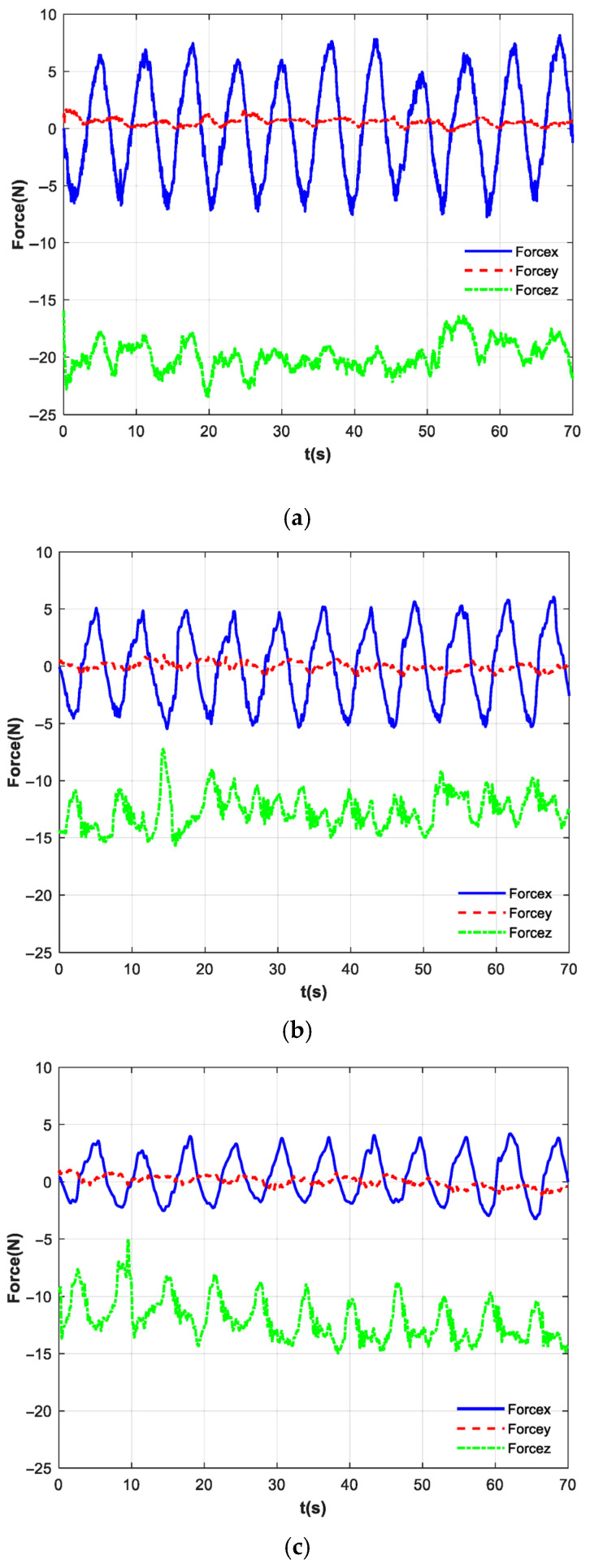
The six-axis force sensor measurement results. (**a**) No feedforward control; (**b**) exponential filter; (**c**) all feedforward control.

The experimental results are shown in Figure 9, Figure 10, Figure 11 and Figure 12. Figure 9 depicts the trajectory of the cup, which is the motion trajectory that assists the subjects in their daily water-carrying rehabilitation training. The red dashed line represents the original motion trajectory of the cup, which corresponds to the motion path of the robot end-effector, while the blue solid line denotes the motion trajectory after processing by the exponential filter. The comparison clearly shows that the filtered trajectory is significantly smoother than the original one. Although the cubic B-spline trajectory is continuous, the exponential filter can further optimize the smoothness of the trajectory, generating more continuous speed and acceleration curves. Furthermore, the exponential filter also possesses low-pass characteristics, enabling it to filter out disruptive components in the trajectory that could potentially excite high-frequency residual vibrations. However, this process inevitably introduces a phase lag.

Figure 10 shows the velocity of the cup. The red dashed line represents the original cup speed (the terminal speed of the robot), and the blue solid line represents the terminal speed after exponential filtering. A comparison of velocity profiles before and after filtering reveals that abrupt velocity changes occur during transitions in the robot end-effector’s trajectory, manifesting as oscillatory behavior around the maximum and minimum values in the plot. These sudden changes cause the ball in the cup–ball system to maintain its original state of motion due to inertia, resulting in persistent vibrations dominated by the natural frequency of the system’s first sloshing mode. By configuring filter parameters aligned with this natural frequency, the exponential filter precisely attenuates vibrational energy at this specific frequency, enabling rapid convergence of residual vibrations and preventing prolonged oscillatory behavior.

Figure 11 represents the robotic-assisted water delivery rehabilitation training experimental results. The blue solid line represents the slosh angle of the ball without feedforward control, with a maximum value of 28.95°, and the red dashed line represents the slosh angle of the ball after the motion trajectory is processed by the exponential filter, with a maximum value of 14.08°, the green center line shows the slosh angle of the ball after all feedforward, with a maximum value of 6.36°. A comparison of the three signals reveals that after adding the exponential filter, the maximum slosh angle of the ball decreases from 28.95° to 14.08°, and the reduction rate of sloshing of the ball is approximately 51.36%. The exponential filter can effectively reduce residual vibration by smoothing the trajectory. However, during the movement of the cup, the slosh angle of the ball is governed by its inertia and the cup’s instantaneous acceleration. A feedforward compensation force is therefore required to effectively suppress the residual sloshing. With the complete feedforward control strategy implemented, the peak slosh angle was reduced to 6.61°, representing a 77.16% reduction. In trials where subjects simulated different levels of participation, the maximum slosh angle exceeded that in fully passive rehabilitation training by 8.35°.

Figure 12 presents the interaction forces between the subject and the handle, as measured by the six-axis force sensor. Figure 12a depicts the interaction forces on the patient’s hand under basic impedance control. The blue solid line represents the force along the *x*-axis, the red dashed line denotes the *y*-axis force, and the green centerline indicates the *z*-axis force. Since the motion trajectory follows the positive/negative x-direction and the subject imitates passive patient movement without applying active force, the robotic arm drives the motion. Consequently, the interaction force in the x-direction opposes the direction of motion displacement. The force range in the x-direction is [−7.74 N, 8.15 N], with a standard deviation of 4.45. There is no movement in the y-direction, and the force is relatively small. Due to the subjects imitating passive training patients, the force in the z-direction is relatively large [−15.91 N, −23.51 N], and the force direction in the *z*-axis is negative all the way downward.

Figure 12b presents the interaction forces between the subject and the handle after incorporating the exponential filter. The blue solid line represents the force along the *x*-axis, the red dashed line denotes the *y*-axis force, and the green centerline indicates the *z*-axis force. The force range in the x-direction is [−5.47 N, 6.05 N], with a standard deviation of 3.32. There is no movement in the y-direction, and the force is relatively small. Due to the subjects imitating passive training patients, the force in the z-direction is [−7.23 N, −15.84 N], and the force direction in the *z*-axis is negative all the way downward. Figure 12c presents the interaction forces between the subject and the handle after all control. The blue solid line represents the force along the *x*-axis, the red dashed line denotes the *y*-axis force, and the green centerline indicates the *z*-axis force. The force range in the x-direction is [−3.22 N, 4.23 N], with a standard deviation of 1.99. The force in the y-direction is also small. The force in the z-direction is [−4.96 N, −14.98 N].

**Figure 13:**
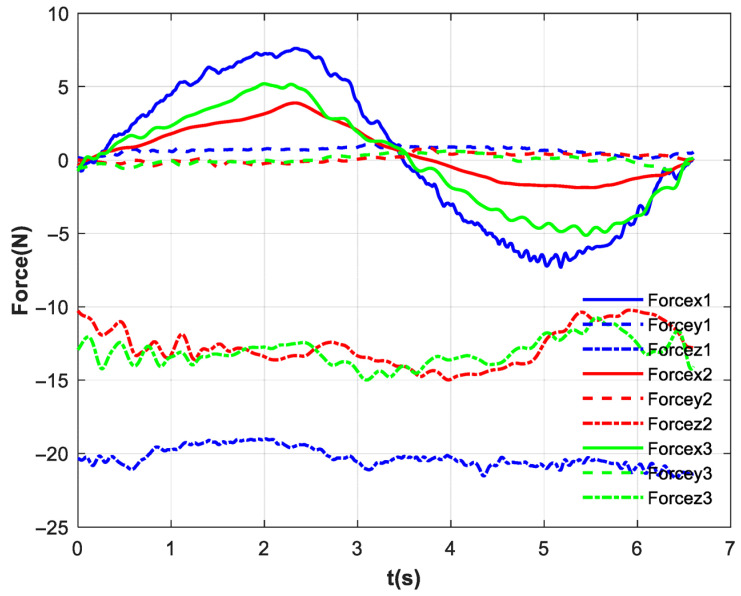
Force in each direction for a single motion cycle.

A comparison of the three figures ‘data reveals that after incorporating the exponential filter, the average interaction force in the X-direction decreased by 2.21 N, and the standard deviation was reduced by 1.36. This indicates that the exponential filtering at the end-effector smoothed high-frequency fluctuations; however, the inertial force of the sphere was not counteracted, and high-frequency disturbances remained noticeable. With the addition of the full feedforward compensation force, the variation in hand forces was reduced by a total of 53.05%, and the standard deviation decreased by 55.28%. The combined strategy demonstrated the most effective improvement in force smoothness. The analysis indicates that the proposed sloshing suppression control strategy can effectively cancel the sphere’s inertial force, suppress high-frequency disturbances, and thereby reduce sphere oscillation. Figure 13 displays the forces in each direction during a single motion cycle. As clearly shown in the figure, the application of the proposed control strategy leads to a lower peak force on the patient’s hand, a marked attenuation of high-frequency oscillations in the force curve, and a more gradual overall trajectory.

### 5.3. Statistical Analysis

Due to the small sample size of the slosh angle, the Kruskal–Wallis test followed by post hoc analysis was used for statistical evaluation. The Kruskal–Wallis test revealed a highly significant overall distribution difference among the three groups (A1, A2, A3) (*p* < 0.0001). The grouping of A1, A2, and A3 has already been described in Figure 11. Subsequent pairwise comparisons using Dunn’s test showed significant differences between A1 and A2 (adjusted *p* = 0.0158), A1 and A3 (adjusted *p* < 0.0001), and A2 and A3 (adjusted *p* = 0.0158). The distribution of mean ranks (A1: 30.50, A2: 18.50, A3: 6.50) clearly indicated a consistent trend of A1 > A2 > A3. This suggests that the indicator level in group A1 was significantly higher than that in groups A2 and A3, while the level in group A2 was significantly higher than that in group A3.

## 6. Discussion

Current rehabilitation training robots primarily employ training paradigms centered on free-space movement training and rigid object interaction. These training environments are characterized by a decontextualized and deterministic nature, failing to address the core challenge of dynamic uncertainties inherent in real-world daily activities. This study proposes a novel rehabilitation training paradigm contextualized around “transporting a water cup”, aiming to enhance patients’ practical ability to safely carry a cup in daily life without spilling the liquid.

Numerous scholars have proposed methods combining feedforward and impedance control, such as model predictive impedance control [22,23], adaptive sliding mode impedance control [20], disturbance observers [21], and adaptive impedance control [19]. However, existing research has primarily focused on the control aspects of human–robot interaction, paying less attention to the internal dynamic variations in the manipulated object. In practical tasks, such as transporting liquid-filled containers, the internal dynamics of the manipulated object often undergo complex changes over time. To address this, our study proposes the establishment of a cup–ball dynamic model and employs a method integrating an exponential filter, compensation force, and impedance control. This approach aims to assist patients in progressively adapting to and enhancing their perception and precise control capabilities in handling uncertain dynamics like liquid sloshing.

Experimental results demonstrate that the exponential filter smooths the robotic trajectory, improving tracking performance and mitigating residual vibrations. The feedforward compensator, derived from the ball’s dynamic model and adaptable to patient needs, estimates and preemptively counteracts inertial disturbance forces to suppress sloshing. Concurrently, impedance control ensures compliant physical assistance, thereby mitigating the risk of secondary injury. The pole of the exponential filter has a negative real part, rendering it asymptotically stable. Its zero-pole cancellation effect helps mitigate the influence of delay. The filter introduces a phase lag of approximately −0.37°, which maintains a phase margin well above the instability threshold, thus having a negligible impact on stability. Although the time delay is close to the sampling period, it does not exceed twice the period, thereby presenting no risk of cumulative delay.

The primary limitation of this study is the evaluation with a small cohort of healthy participants rather than the target patient population (e.g., stroke or hemiplegic patients). While this provides valuable proof-of-concept and initial validation of the system’s functionality and safety, future studies with clinical populations are necessary to validate its therapeutic efficacy.

## 7. Conclusions

The ability of stroke patients to interact with complex everyday objects is crucial for rehabilitation. This paper investigates the use of a rehabilitation robot to assist patients in transporting a water cup, with the goal of enhancing their perception and control of liquid sloshing dynamics. We designed a cup–ball system compatible with a rehabilitation robotic arm and proposed a sloshing-suppression control strategy integrating exponential filtering, feedforward compensation, and impedance control.

The results demonstrate that the integrated control strategy outperforms any single control method. Sloshing of the ball amplitude and the standard deviation of the human–robot interaction force were both significantly reduced, effectively eliminating inertial shaking caused by instantaneous acceleration and residual end-point vibration. These outcomes meet the requirements for smoothly transporting a cup of water in a rehabilitation context. The proposed method represents an advancement from traditional position-tracking strategies to a dynamic disturbance-suppression paradigm, thereby improving the patient’s ability to perceive and control liquid motion.

Future research should aim to move beyond the current simplified rigid-body spherical pendulum model. It is crucial to develop a multi-physics, nonlinear dynamic model that incorporates flexible deformations. Furthermore, intelligent control strategies should be optimized by integrating impedance control with reinforcement learning, thereby enhancing individual adaptability and sway suppression precision.

## Data Availability

The original contributions presented in this study are included in the article. Further inquiries can be directed to the corresponding author.
